# Editorial: Leukocyte Trafficking in Homeostasis and Disease

**DOI:** 10.3389/fimmu.2019.02560

**Published:** 2019-11-01

**Authors:** Joaquin Teixidó, Andres Hidalgo, Susanna Fagerholm

**Affiliations:** ^1^Department of Molecular Biomedicine, Centro de Investigaciones Biológicas (CSIC), Madrid, Spain; ^2^Department of Cell and Developmental Biology, Centro Nacional de Investigaciones Cardiovasculares, Madrid, Spain; ^3^Institute for Cardiovascular Prevention, Ludwig-Maximilians University, Munich, Germany; ^4^Research Program of Molecular and Integrative Biosciences, Faculty of Bio- and Environmental Sciences, University of Helsinki, Helsinki, Finland

**Keywords:** leukocyte, traffic, homeostasis, inflammation, cancer

Leukocytes move avidly through the body. While this is classically associated with immune responses, leukocyte trafficking is just as prominent during steady-state conditions as they leave the bone marrow (BM), home back to tissues for elimination, or traffic through secondary lymphoid organs (1). However, immune cell trafficking becomes uncontrolled during inflammatory pathologies (2, 3), and in the homing of hematologic tumor cells to BM and lymph nodes. Diapedesis of immune cells and blood cancer cells across endothelium is facilitated by chemokines and adhesion molecules, which act in concert in tightly regulated directional motility (1–6).

The Research Topic on “Leukocyte Trafficking in Homeostasis and Disease” covers several reviews providing an up-to-date view of different molecular and cellular players that regulate key trafficking processes during cell differentiation, immune responses and lymphocyte recirculation, as well as in inflammatory pathologies and in hematological malignancies.

## Neutrophil Trafficking

Integrins are key adhesion receptors controlling leukocyte trafficking. In their review, Fagerholm et al. describe the critical roles of β2 integrins in leukocyte trafficking and other leukocyte functions that are dependent on cell adhesion (7). The importance of β2 integrins for immune function is shown by rare genetic disorders (Leukocyte adhesion deficiencies, or LAD) that affect their expression (LAD-I) or function (LAD-III, caused by mutations in the integrin regulator, kindlin-3) (8, 9). However, β2 integrins are also associated with many immune suppressive functions. For example, they can inhibit tissue migration of dendritic cells, and also suppress cytokine responses in myeloid cells (10–12). Because of these various roles in immunity, β2 integrin dysfunction can contribute to the development of both immunodeficiency diseases and inflammatory diseases.

The classical leukocyte recruitment cascade consisting of leukocyte capture, rolling, arrest, firm adhesion, crawling and finally, transmigration through the endothelium (2), is widely accepted as the way leukocytes are recruited into tissues. Maas et al. describe the variations in the trafficking rules that neutrophils use to enter different tissues, focusing on lung, liver, kidney and aorta. These rules are distinct in different organs and tissues, and there appears to be significant redundancy in the system (chemokines, etc.), which may explain why it is so difficult to target leukocyte recruitment successfully in the clinic.

Focusing on the migratory dynamics of neutrophils, Morikis and Simon summarize an extensive body of literature suggesting that biomechanical signals at the original site of interactions between leukocytes and the activated endothelium critically regulate leukocyte adhesion and polarization. The extensive, but poorly characterized interplay between multiple types of receptors (selectins, glycoproteins, integrins, or calcium channels) in these regions is discussed to be critical at these early stages of the recruitment cascade. With the description of how protein modules in integrins and cytoskeleton reorganize, and the relevance of these events in controlling neutrophil migration, Morikis and Simon provide an exciting review of the intricate biomechanics of immunity.

Neutrophils must cross multiple barriers in the body to reach the areas where they will perform their immune tasks (13). An underappreciated aspect of this migration is how the cell adapts to the constraints imposed by each barrier, be it endothelial, matricial, or interstitial. Salvermoser et al. discuss how neutrophils adapt to the varying environments, and in particular focus on adaptations of nucleus, which is the stiffest cellular organelle. As thoroughly reviewed by the authors, nuclear architecture and deformability are key features that allow the swift and efficient migration of neutrophils through multiple environments.

Leukocytes not only travel into peripheral tissues, but can interestingly also regulate BM hematopoietic stem cells (HSC) (14). The review by Lucas describes the HSC niche, its components and its regulation by leukocytes and by leukocyte trafficking. HSCs give rise to leukocyte subtypes (including neutrophils), which feed back to the HSC niche, regulating both HSC number and function. This crosstalk may function as a biological rheostat during inflammation and in different disease states, and this feedback system allows the BM to monitor the periphery and to adjust leukocyte output according to peripheral needs, although many of the finer details still remain to be elucidated.

## Platelets in Leukocyte Recruitment and Resolution of Inflammation

Current research has expanded the appreciation of platelets beyond their contribution to primary hemostasis, indicating that they also actively participate in leukocyte recruitment, especially neutrophils, and in the regulation of the host defense in response to exogenous injuries (15). Platelets physically interact with different leukocyte subsets during inflammatory processes (16), which hold extensive implications for the leukocyte recruitment into peripheral tissues and for the regulation of leukocyte cell autonomous functions, including the formation and liberation of neutrophil extracellular traps. In addition, platelets have also been implicated in the resolution of inflammation (17). The review by Rossaint et al. focuses on the role of platelets in leukocyte recruitment during the initiation of the host defense, and also discusses their participation in the resolution process after acute inflammation.

## Monocyte and Macrophage Trafficking

Teh et al. describe recent advances in the field of monocyte trafficking. Monocytes are highly plastic cells which can perform effector functions in their own right, or traffic into tissues and differentiate into various monocyte-derived cell types, both during homeostasis and in different diseases (18). Major advances in understanding the role of monocytes and monocyte-derived cells were possible in recent years due to development of imaging techniques, but the authors point out that these cells are still challenging to investigate due to their plasticity.

In an original research paper included in this Topic, Cui et al. studied the role of the αLβ2 and αDβ2 integrins in macrophage migration in tissues. They show that 3D amoeboid macrophage migration is inhibited by high β2 integrin expression, whilst a moderate expression of the integrin promotes it.

## Catecholamines, Glucocorticoids and Scavenger Receptors

Ince et al. provide an exhaustive overview of the dynamics of multiple leukocyte subsets with particular emphasis on the molecular cues guiding their trafficking patterns during baseline or inflammatory conditions. The first of these cues are catecholamines, which are neurotransmitters produced by the adrenal gland, sympathetic nerves and even leukocytes themselves. Recent studies established important roles for catecholamines in regulating the expression of adhesion molecules and chemoattractants by endothelial cells, but also through direct actions on leukocytes (19, 20). A second class of cues is the glucocorticoids, a type of steroid hormones produced by the adrenal gland. These hormones influence many aspects of leukocyte behavior by generally reducing their adhesive capacity (21, 22). This can induce, for example, potent demargination of certain leukocyte types by attenuating interactions with vascular cells (23). Because the presence of both signals display potent circadian patterns, the review also discusses how these signals contribute to diurnal rhythms in leukocyte trafficking.

The review by Patten and Shetty describes roles of scavenger receptors expressed on endothelial cells (24), which regulate the leukocyte trafficking. However, the roles of these receptors in leukocyte migration are less well-understood than those of other traditional adhesion receptors.

## Hematologic Tumor Cell Trafficking

The trafficking of hematologic tumor cells is facilitated by adhesion molecules and chemokines, a process that contributes to progression of hematologic malignancies (4, 6). A common feature of multiple myeloma, chronic lymphocytic leukemia and acute lymphoblastic leukemia is the homing and lodging of blood cancer cells in the BM, which favors their growth and survival. The α4β1 integrin and the chemokine receptor CXCR4 are key molecules for cell trafficking into and out of the BM in these hematologic neoplasias (25, 26). Redondo-Muñoz et al. review the molecular players that regulate the trafficking of neoplastic cells during development and progression of these hematologic malignancies.

## Author Contributions

All authors listed have made a substantial, direct and intellectual contribution to the work, and approved it for publication.

### Conflict of Interest

The authors declare that the research was conducted in the absence of any commercial or financial relationships that could be construed as a potential conflict of interest.
